# The relationship between physical activity, sleep, and negative emotions in physically weak college students

**DOI:** 10.3389/fpubh.2025.1530988

**Published:** 2025-07-23

**Authors:** Yunchen Meng, Yang Wang, Qiushi Liu, Chao Liang, Sanjun Yang

**Affiliations:** ^1^Department of Physical Education and Research, China University of Mining and Technology, Beijing, China; ^2^RDFZ Chaoyang School, Beijing, China; ^3^Beijing National Day School-Zhong Guan Cun Ke Xue Cheng, Beijing, China

**Keywords:** physically weak college students, physical activity, depression, anxiety, stress, sleep quality

## Abstract

**Background:**

Negative emotions among college students, particularly those who are physically weak, have raised concerns due to their impact on mental health and, in some cases, the development of suicidal tendencies. Limited physical activity exacerbates these mental health risks. It is essential to explore the relationship between physical activity, sleep quality, and negative emotions in this population.

**Objective:**

To examine the relationship between physical activity, sleep quality, and negative emotions among physically weak college students.

**Methods:**

A stratified sampling approach was employed to select 1,248 first- and second-year students from a university in Beijing. Physical activity levels were assessed using the International Physical Activity Questionnaire Short Form (IPAQ-SF), sleep quality was evaluated with the Pittsburgh Sleep Quality Index (PSQI), and depression, anxiety, and stress were measured via the Depression Anxiety Stress Scales (DASS-21). Chi-square tests and logistic regression analyses were conducted to explore associations among these variables. Additionally, a mediation effect model was utilized to examine the mediating role of sleep quality between physical activity and negative emotions.

**Results:**

The incidence of negative emotions was significantly higher among physically weak college students compared to their peers (*p* < 0.05). Binary logistic regression results indicated that increased physical activity intensity was associated with a lower occurrence of negative emotions (*OR* = 0.514, *95% CI*: 0.367–0.719). Linear regression analysis showed a significant positive correlation between higher physical activity intensity and better sleep quality (*a* = −0.185, *p* < 0.01). The mediation effect analysis revealed that sleep quality partially mediated the relationship between physical activity and negative emotions, with total, direct, and indirect effects of −0.67, −0.60, and −0.097, respectively. Sleep quality accounted for 14.6% of the total effect in this relationship.

**Conclusion:**

The incidence of negative emotions among physically weak college students is significantly higher than among their peers and increased physical activity intensity is significantly associated with a decrease in negative emotions. Sleep quality plays a crucial mediating role in this relationship. These findings provide a theoretical foundation for developing targeted mental health interventions for physically weak college students and highlight the importance of sleep quality in improving their mental health.

## 1 Introduction

Negative emotions experienced by college students constitute significant factors influencing their mental health (1). As a critical demographic navigating the transition from adolescence to adulthood, college students undergo profound physiological, psychological, and social transformations. This period is often deemed one of the most challenging times in life, as students frequently grapple with multiple stressors, including academic pressures, interpersonal relationships, and career planning (2). Negative emotions represent adverse attitudinal experiences and corresponding behavioral responses to objective phenomena, often triggering intense physiological and behavioral changes that manifest as various emotional states, such as tension, sadness, fear, guilt, anger, contempt, and disgust (3). Anxiety and depression are particularly prevalent negative emotions among college students. Extensive research has shown that when individuals are unable to adapt to environmental demands, the resulting stress can foster negative emotions and pessimistic beliefs, leading to the rapid onset of anxiety and depression symptoms under pressure (4). Alarmingly, data from the American Psychological Association (APA) reveals that over 60% of college students were diagnosed with at least one mental health issue during the 2020–2021 academic year, underscoring the dire state of mental health among this population (5). Similarly, mental health concerns among college students in China are pressing, with the “China National Mental Health Development Report (2021–2022)” indicating that the detection rate of depressive mood among Chinese college students reached 10.6%, and the risk detection rate for anxiety soared to 15.8%. Notably, the depression risk detection rate for the 18–24 age group stood at 24.1%, significantly higher than that of other age groups (6). These data unequivocally emphasize the gravity of mental health issues among college students, necessitating our utmost attention to their mental wellbeing. Identifying effective predictors of mental health is crucial for effectively preventing the emergence of negative emotions in this vulnerable population.

Physically weak college students constitute a distinct group that encounters various challenges, such as physical disabilities, chronic illnesses, obesity, or underweight, often leading to poor physical fitness and limited participation in routine activities (7). This condition not only hampers their daily functioning but also renders them more susceptible to emotional fluctuations and negative psychological states (8). Negative emotions, including depression, anxiety, and stress, can severely impair an individual’s mental health, subsequently affecting their physical well-being and academic performance, thereby perpetuating a vicious cycle (9).

A wealth of research has demonstrated that physical exercise plays a pivotal role in mitigating negative emotions (10, 11). As a vital and efficacious method for promoting physical health, physical exercise also serves as a green and healthy intervention to effectively prevent aggressive behavior among college students (12). According to the frustration-reaction theory, individuals may experience a series of negative psychological states when they encounter obstacles that hinder them from achieving their goals or satisfying their motivations. By increasing the frequency and intensity of physical exercise, individuals’ psychological resilience can be significantly bolstered, actively promoting the improvement of mental health and providing robust theoretical support for maintaining a positive psychological state (13). Furthermore, moderate aerobic exercise has been shown to reduce negative emotional reactions, with particularly pronounced effects among those who have difficulty regulating their emotions (14). Research has indicated that regular physical activity not only enhances physical fitness but also promotes the release of endorphins and other neurotransmitters in the brain, which can uplift mood and alleviate symptoms of anxiety and depression (15). However, within the broader college population, physically weak students encounter particularly pronounced mental health challenges. Due to their physical limitations, they may struggle to engage in physical activities as actively as their peers, despite the widely acknowledged effectiveness of physical activity in alleviating stress and enhancing mood.

Moreover, physical activity contributes to better sleep quality, a vital factor in maintaining mental health (16). The well-established relationship between sleep quality and mental health is supported by the circadian rhythm theory, which posits that the human body possesses an endogenous biological rhythm system that regulates the cyclical changes of physiological and behavioral activities over a 24-h period (17). Poor or inadequate sleep can adversely affect cognitive functions and emotional regulation, increasing the incidence of issues such as depression and anxiety (18). At the neurobiological level, sleep and emotion regulation share common mechanisms. Studies have shown that daytime events significantly influence sleep, and the quality of nighttime sleep indirectly affects an individual’s emotional response to subsequent events (19, 20). For physically weak college students, sleep disorders stemming from physical issues may be more severe, further exacerbating their mental health burdens. Therefore, investigating the interrelationships between physical activity, sleep quality, and negative emotions among physically weak college students is essential for developing effective mental health intervention strategies. However, physically weak college students often lack adequate physical activity due to their health conditions, resulting in poorer sleep quality and an elevated risk of negative emotions.

This study aims to compare the differences in physical activity, sleep quality, and negative emotions between physically weak college students and their healthier counterparts, exploring the underlying connections among these variables. We hypothesize that physically weak college students will demonstrate significantly lower levels of physical activity and poorer sleep quality, leading to a higher prevalence of negative emotions. By quantifying these data, we aspire to gain a clearer understanding of the mental health status and influencing factors affecting physically weak college students, providing a scientific basis for targeted mental health interventions. Furthermore, these research findings hold significant potential for application in university counseling centers, physical education departments, and online health intervention platforms, enabling the creation of more tailored activity plans, personalized counseling programs, and digital health management tools, ultimately aiming to improve the overall health and well-being of this specific group.

## 2 Methods

### 2.1 Participants

This study employed a stratified random sampling approach to select students from general physical education courses, physical fitness enhancement programs, and sports rehabilitation classes among first- and second-year students at a university located in Haidian District, Beijing. All participants voluntarily engaged in the study and, after providing informed consent, were guided by trained personnel to complete the questionnaires. A total of 1,475 questionnaires were collected, with 1,248 deemed valid after excluding incomplete and invalid responses, resulting in an effective recovery rate of 84.6%. The sample comprised 632 males (50.64%) and 616 females (49.36%), with 636 first-year students (50.96%) and 612 s-year students (49.04%). Additionally, 1,040 participants were enrolled in general physical education courses (83.33%), 116 in physical fitness enhancement programs (9.29%), and 92 in sports rehabilitation classes (7.37%). The average age of the respondents was 19.33 ± 0.87 years.

### 2.2 Measurement tools

#### 2.2.1 Basic information

The questionnaire gathered demographic data from university students, encompassing gender, age, ethnicity, height, weight, participation in physical education courses, and detailed health status information for students in rehabilitation classes.

#### 2.2.2 Physical activity

The study utilized the International Physical Activity Questionnaire Short Form (IPAQ-SF) to gather data on physical activity levels among university students. The IPAQ-SF is widely recognized and extensively used in public health and epidemiological research (21). The questionnaire comprises seven concise questions that encompass vigorous activity, moderate-intensity activity, walking, and sedentary time, designed to gather information on the number of days and the specific duration spent on each activity category over the past 7 days. Following data collection, rigorous cleaning and processing were conducted in accordance with the IPAQ guidelines (22): (1) Cases where the total sum of moderate-intensity and above physical activities, walking, and time variables exceeded 960 min per day were excluded; (2) Any segment of vigorous activity, moderate-intensity activity, and walking time that surpassed 180 min per day was truncated to 180 min per day. Afterwards, the weekly energy expenditure was calculated based on the IPAQ-SF scoring guidelines, and individuals’ physical activity intensity was classified into low, moderate, and high levels.

#### 2.2.3 Sleep quality

The Pittsburgh Sleep Quality Index (PSQI) was used to evaluate participants’ sleep quality. The PSQI is a self-assessment questionnaire designed to comprehensively evaluate an individual’s sleep quality and potential sleep disturbances that may have occurred over the past month (23). The questionnaire consists of 18 items, which can generate 7 component scores, each ranging from 0 to 3. These components include subjective sleep quality, sleep latency, sleep duration, sleep efficiency, sleep disturbances, use of sleep medications, and daytime dysfunction. These 7 component scores are ultimately summed into a global score, ranging from 0 to 21, with higher scores indicating poorer sleep quality. A score exceeding 5 indicates poor sleep quality (24).

#### 2.2.4 Depression anxiety stress scales

This Depression Anxiety Stress Scales-21 (DASS-21) was applied to assess negative emotional states among university students. Widely employed in both clinical and research settings (25, 26), the DASS-21 comprises 21 items divided into three dimensions: depression, anxiety, and stress, with each dimension containing seven items. Each item is rated on a 4-point Likert scale to reflect the extent to which the statement applies or is true for the respondent over the past week. A higher total score indicates more severe negative emotions in the respondent. Scores for each dimension range from 0 to 21, with ≥10 indicating depressive symptoms, ≥8 indicating anxiety symptoms, and ≥15 indicating excessive stress (27).

### 2.3 Quality control

To ensure the high quality of the research and the precision of the data, a comprehensive standardization of the research plan and survey execution has been implemented. Prior to the commencement of the survey, systematic training was provided to the teachers acting as surveyors, encompassing the formulation of standardized introductions, thorough familiarization with the questionnaire content, and meticulous attention to various details pertinent to questionnaire completion. Furthermore, a rigorous set of data cleaning protocols has been established to uphold the external validity of the data subjected to analysis. During the data preprocessing phase, entries will be scrutinized for logical inconsistencies, missing data, inaccuracies, or ambiguous responses, and re-testing or exclusion will be undertaken as necessary, to uphold the authenticity and validity of the dataset. Researchers adopted a stratified sampling method for course selection in the entire process of conducting a questionnaire survey among college students. The main investigators consistently upheld and emphasized the anonymity and confidentiality principles of the questionnaire. They clearly explained to the respondents that the collected data would be used solely for scientific research purposes, and they made every effort to minimize various biases that might arise during the standardized survey process, in order to ensure the objectivity, fairness, and reliability of the survey results.

### 2.4 Data analysis

Statistical analysis was conducted using SPSS 26. Quantitative data were presented as *M ±* SD, while categorical data were expressed as *n* (%). Chi-square tests were employed to analyze differences between groups. Physical activity was treated as a binary independent variable (*X*), sleep quality as a mediating variable (*M*), and negative emotions as a binary dependent variable (*Y*). The R software’s RMediation package was utilized for the analysis. All continuous variables were standardized to mitigate the influence of course variables. Logistic regression models were sequentially applied to assess the associations among physical activity, sleep quality, and negative emotions, while linear regression calculated the relationship between physical activity and sleep quality. The significance of the mediation effect was tested using the bias-corrected bootstrap method with 5,000 iterations.

## 3 Results

### 3.1 Basic characteristics of the survey participants

Before examining the mechanisms through which physical activity influences negative emotions in college students, this study first outlined the basic characteristics of each variable, including sample size, percentage distribution, and chi-square statistics (Table 1). The descriptive analysis of the surveyed university students revealed that 208 students (16.7%) were classified as having poor physical health, primarily drawn from physical fitness enhancement and sports rehabilitation courses. The BMI distribution was as follows: 86 students (6.9%) were underweight, 806 students (64.6%) had a normal weight, 171 students (13.7%) were overweight, and 185 students (14.8%) were obese. Regarding PA levels, the survey indicated that 280 students (22.4%) engaged in low-level physical activity (LPA), 672 students (53.9%) participated in moderate-level physical activity (MPA), and 296 students (23.7%) engaged in vigorous-level physical activity (VPA). Sleep quality assessments showed that 528 students (42.3%) reported good sleep quality, while 720 students (57.7%) experienced poor sleep quality, which constituted a significant proportion of the sample. In terms of negative emotions, 201 students (16.1%) exhibited depressive symptoms, 402 students (32.2%) demonstrated anxiety symptoms, and 137 students (11.2%) reported stress symptoms. The chi-square test revealed a statistically significant higher prevalence of depression and anxiety among students with poor physical health compared to their healthier peers (*p* < 0.05). Additionally, students engaging in low levels of physical activity displayed a significantly higher incidence of depression and anxiety than those maintaining moderate to high levels of physical activity (*p* < 0.05). Furthermore, students with poor sleep quality were more likely to experience negative emotions than those with good sleep quality (*p* < 0.05).

**Table 1:** Basic characteristics of survey respondents.

Characteristics	Sample size	Depression				Anxiety				Stress			
		*N*	%	*X* ^2^	*p*	*N*	%	*X* ^2^	*p*	*N*	%	*X* ^2^	*p*
Gender													
Male	632	126	19.9	13.91	<0.001	213	33.7	1.30	0.254	95	15.0	18.70	<0.001
Female	616	75	12.2			189	30.7			45	7.3		
Grade													
First	636	88	13.8	18.70	<0.001	210	33.0	0.39	0.534	72	11.3	0.01	0.907
Second	612	113	18.5			192	31.4			68	11.1		
Nation													
Han	1,127	183	16.2	0.15	0.699	354	31.4	3.41	0.065	130	11.5	1.17	0.279
Others	121	18	14.9			48	39.7			10	8.3		
P. E. Course													
General	1,040	151	14.5	13.80	0.001	318	30.6	9.73	0.008	118	11.3	1.11	0.574
Enhancement	116	24	20.7			42	36.2			10	8.6		
Rehabilitation	92	26	28.3			42	45.7			12	13.0		
BMI													
Low	86	24	27.9	16.17	0.001	36	41.9	8.77	0.033	13	15.1	2.94	0.402
Normal	806	113	14.0			242	30.0			82	10.2		
Overweight	171	24	14.0			53	31.0			22	12.9		
Obesity	185	40	21.6			71	38.4			23	12.4		
PA													
LPA	280	66	23.6	14.89	<0.001	110	39.3	8.90	0.012	32	11.4	0.79	0.674
MPA	672	94	14.0			208	31.0			71	10.6		
VPA	296	41	13.9			84	28.4			37	12.5		
Sleep quality													
Good	528	49	9.3	31.56	<0.001	82	15.5	116.63	<0.001	36	6.8	17.79	<0.001
Poor	720	152	21.1			320	44.4			104	14.4		

### 3.2 Mediating effect of sleep quality in the relationship between physical activity and negative emotions

The mediating effect of sleep quality in the relationship between physical activity and negative emotions was further explored. After controlling for the variable of physical education courses, the binary logistic regression analysis was conducted. Model fit was assessed using the Hosmer-Lemeshow test, which indicated a strong fit (*X^2^* = 0.635, *p* = 0.728), demonstrating that the model reliably predicts the observed outcomes. Notably, the study found that students engaging in moderate to vigorous physical activity (MVPA) had a significantly lower risk of negative emotions compared to those with LPA. The odds ratio (*OR*) for this comparison was 0.514, with a 95% confidence interval ranging from 0.367 to 0.719. Furthermore, the total effect of physical activity on negative emotions, mediated through sleep quality, was significant (*C* = −0.666, *p* < 0.001), underscoring the critical role of sleep quality as a mediator.

Linear regression analysis revealed a significant positive correlation between higher physical activity intensity and better sleep quality, with a correlation coefficient (*a*) of −0.185 (*p* < 0.01). To further investigate the interplay between physical activity, sleep quality, and negative emotions, a binary logistic regression model was employed, which demonstrated excellent fit as confirmed by the Hosmer-Lemeshow test (*X^2^* = 11.634, *p* = 0.168). The direct effect of physical activity on negative emotions was also statistically significant (*c’* = −0.602, *p* < 0.01), highlighting the protective role of physical activity against negative emotions. Additionally, the regression coefficient for the relationship between sleep quality and negative emotions was *b* = 0.527 (*p* < 0.001), emphasizing the critical influence of sleep quality on emotional well-being (Table 2).

**Table 2:** A logistic regression analysis examining the associations among physical activity, sleep quality, and negative emotions.

Index	*β*	SE	*Z*	*p*	OR	95%CI	
Independent variable							
Constant	−1.455	0.154	−9.433	0.000	0.233		
PA (MVPA→LPA) (c’)	−0.602	0.176	−3.428	0.001	0.548	0.389, 0.776	
Sleep quality (b)	0.527	0.077	6.837	0.000	1.694	1.458, 1.974	
Control variables							
Course 1 (enhancement→general)	0.729	0.256	2.843	0.004	2.073	1.234, 3.383	
Course 2 (rehabilitation→general)	0.886	0.259	3.423	0.001	2.424	1.441, 3.986	
*X* ^2^	11.634						
*p*	0.168						

**Figure 1:**
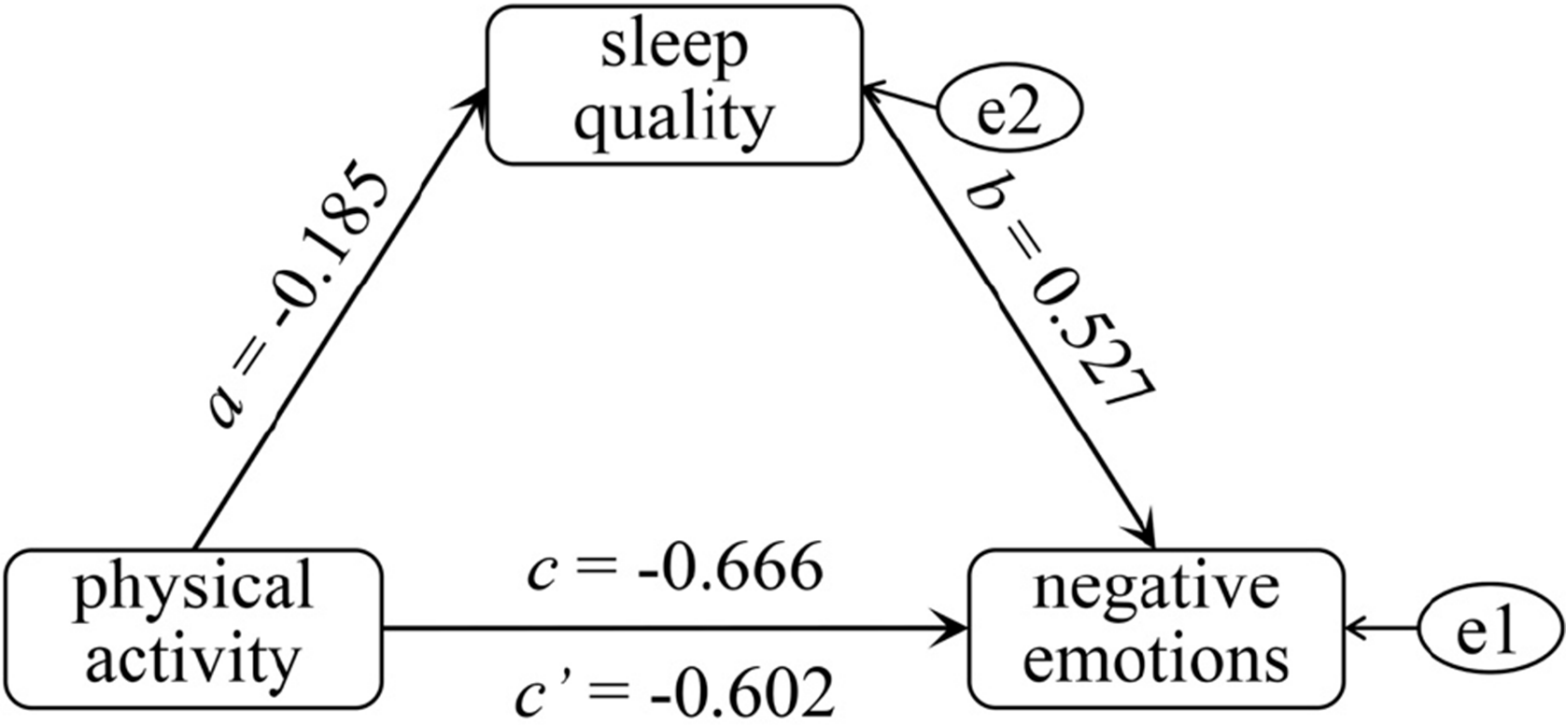
The pathway of sleep quality’s influence on the relationship between physical activity and negative emotions.

The mediation effect of sleep quality on the relationship between physical activity and negative emotions was further evaluated using the bootstrap method with 5,000 iterations to ensure robustness. Results indicated that sleep quality significantly mediated this relationship, with a mediation effect estimate (*β*) of −0.097. The 95% confidence interval for this estimate ranged from (BootLLCI) -0.185 to (BootULCI) -0.026, confirming the statistical significance of the mediation effect. This mediation effect accounted for 14.6% of the total effect, thereby elucidating a distinct pathway through which physical activity exerts its influence on negative emotions, with sleep quality serving as a crucial mediator (Figure 1).

## 4 Discussion

This study explored the complex relationships between physical activity, sleep quality, and negative emotions among physically weak college students. It confirmed that these students experience significantly higher levels of negative emotions compared to their healthier counterparts. Moreover, the study revealed a significant negative correlation between the intensity of physical activity and negative emotions. Additionally, it identified a partial mediating role of sleep quality in the relationship between physical activity and negative emotions, offering new insights into the mechanisms through which physical activity influences mental health in college students. Specifically, physical exercise was found to reduce negative emotions among physically weak college students, with sleep quality serving as a partial mediator in this process. These findings highlight the indirect yet important role of sleep quality in enhancing the mental health benefits of physical exercise, providing valuable scientific evidence for the development of targeted mental health intervention strategies for physically weak college students.

### 4.1 Basic conditions and mental health issues of students with poor physical health

This study offers valuable insights into the physical activity, sleep quality, and mental health status of university students with poor physical health, shedding light on the unique psychological challenges faced by this group. Our findings revealed that 16.7% of surveyed students reported poor physical health, a figure lower than the 30% failure rate documented in recent years (28), yet still significant enough to warrant attention. In terms of physical activity, 22.4% of students with poor physical health exhibited low activity levels, a proportion notably higher than that observed among their healthier peers. Additionally, 57.7% of this group reported poor sleep quality, far surpassing the average levels among typical university students. These deficits in physical activity and sleep quality contribute to an increased mental health risk for students with poor physical health. Regarding mental health, the prevalence of negative emotions such as depression, anxiety, and stress was significantly higher among students with poor physical health. Specifically, 36.6% of these students reported experiencing high levels of negative emotions, a rate comparable to the detection rates of depression and anxiety among university students in the 2022 edition of the “National Mental Health Report of China” (29). Moreover, within physical enhancement and sports rehabilitation classes, the proportions of students experiencing negative emotions were as high as 41.4 and 50%, respectively, further highlighting the vulnerability of students with poor physical health to mental health issues. These students’ susceptibility to mental health problems is further exacerbated by academic pressures, social challenges, and life stressors stemming from their physical condition (16). Their insufficient physical activity and poor sleep quality not only directly impair their physical health but also indirectly contribute to the development of negative emotions. Consequently, it is crucial to implement proactive and effective interventions to address the mental health issues of students with poor physical health. These interventions should focus on enhancing physical activity levels, improving sleep quality, and alleviating negative emotions, ultimately promoting both physical and mental well-being.

### 4.2 The relationship between physical activity and negative emotions

Through regression model analysis, this study has illuminated the complex relationship between physical activity intensity and negative emotions among university students. After controlling for the variable of physical education courses, we found that increased physical activity intensity significantly reduced the incidence of negative emotions. This finding aligns with existing research, underscoring the importance of physical activity in maintaining psychological well-being (3). The rapid advancements in science and technology in modern society have brought about significant changes in lifestyle and work patterns, with insufficient physical activity emerging as a major public health concern (30). This issue is particularly acute for students with poor physical health. Research indicates that a lack of physical activity not only impairs physical health but also has profound effects on mental health, heightening the risk of negative emotions such as depression, anxiety, and stress (31). Due to their physical limitations and insufficient activity, students with poor physical health are more vulnerable to emotional difficulties.

Engaging in regular moderate to vigorous physical activity is especially critical for these students. Such activities not only enhance physical fitness and improve bodily functions but also alleviate psychological stress by fostering social interactions, regulating neuroendocrine functions, and improving overall emotional health (32). Increased social interaction helps reduce feelings of loneliness and social anxiety, while neuroendocrine regulation balances hormone levels, promoting emotional stability. Moreover, meta-analyses have shown that weekly engagement in moderate to vigorous physical activity significantly lowers the risk of depression (33), further reinforcing the positive impact of physical activity on mental health. Additionally, the regression analysis revealed a significant role of sleep quality in influencing negative emotions among university students. Insufficient sleep can lead to physiological dysfunction, decreased attention and memory, and emotional instability, thereby exacerbating negative emotions (34). For students with poor physical health, good sleep quality is essential not only for physical health but also for psychological well-being. Therefore, in promoting physical activity among these students, it is equally important to prioritize improving their sleep quality, thereby creating a comprehensive psychological health support system.

### 4.3 Mediating effects of sleep quality

T The mediation effect analysis in this study further clarified the intricate interplay between sleep quality, physical activity, and negative emotions. The results demonstrated that sleep quality serves as a mediating variable in the relationship between physical activity and negative emotions, significantly influencing the overall effect of this interaction. This finding provides a novel perspective on maintaining mental health among students with poor physical health. Moderate to vigorous physical activity directly enhances emotional well-being by regulating neurotransmitters and stress hormone levels, thereby alleviating negative emotions such as depression and anxiety (35). This physiological regulatory mechanism underscores the critical role of physical activity in supporting mental health. Additionally, physical activity indirectly influences emotional states by inducing a moderate level of fatigue, which facilitates better sleep, further contributing to mental health improvements (36).

The importance of sleep quality in emotional stability and mental health cannot be overstated. High-quality sleep promotes brain function restoration, enhances the immune system, and regulates hormone levels, all of which are essential for emotional regulation and stability (37). Numerous studies have identified sleep quality as a significant mediating factor for mental health, closely linked to the occurrence of negative emotions (38, 39). This study found that physical activity alleviated negative emotions among students with poor physical health by improving sleep quality, consistent with existing literature. Moreover, a synergistic relationship between regular moderate to vigorous physical activity and good sleep was observed. Physical activity enhances feelings of fatigue, facilitating the onset and maintenance of deep sleep, while good sleep provides the energy and recovery needed for sustained physical activity. This positive feedback loop underscores the combined importance of physical activity and sleep quality in maintaining mental health (40).

### 4.4 Applications and practical implications

The findings of this study offer valuable insights into addressing the mental health challenges faced by university students with poor physical health. University mental health support services should prioritize this vulnerable group by providing personalized counseling, sleep management training, and targeted psychological interventions aimed at reducing stress and enhancing emotional stability. Additionally, campus health and fitness programs should incorporate tailored physical activities suitable for these students, such as yoga, tai chi, swimming, or group walking exercises. These activities not only improve physical fitness but also enhance sleep quality and regulate neurotransmitter levels, ultimately contributing to better mental health outcomes. Furthermore, universities should consider developing digital health management platforms that integrate data from wearable devices to monitor students’ physical activity and sleep patterns. These platforms can provide personalized recommendations to encourage healthier lifestyle choices. On a broader scale, university administrators should implement flexible course schedules and adaptive academic policies to alleviate academic pressure and create a more inclusive learning environment for students with poor physical health. Beyond the university setting, community health service centers can apply these findings to support broader populations of young adults with poor physical health. Interventions focused on increasing physical activity and improving sleep quality could help mitigate the risk of mental health issues. These practical implications emphasize the importance of comprehensive support measures in fostering a healthier and more supportive environment, ultimately improving the overall quality of life for students with poor physical health.

## 5 Limitations and future directions

This study highlights the relationship between physical activity, sleep quality, and negative emotions in physically weak college students, emphasizing sleep quality’s mediating role. However, limitations include a single-university sample, reliance on self-reported data, a cross-sectional design, and unaccounted confounding variables. Future research should involve more diverse samples, objective measures, longitudinal designs, and additional influencing factors. Expanding beyond physically weak students, studies could explore tailored interventions combining physical activity and sleep strategies, utilize digital health tools, and compare various student populations. These findings can guide university policies to enhance student well-being and evaluate their effectiveness.

## 6 Conclusion

Students who experience poor physical health exhibit notably elevated levels of inadequate physical activity, suboptimal sleep quality, and adverse emotional states when compared to their healthier counterparts, highlighting their heightened vulnerability to mental health concerns. Engaging in increased physical activity has a direct impact on diminishing negative emotions, with moderate-to-vigorous activities demonstrating particular efficacy in alleviating symptoms of depression, anxiety, and stress. Additionally, sleep quality acts as a mediator between physical activity and negative emotions; quality sleep further amplifies the reduction of such emotional distress. Consequently, prioritizing the enhancement of physical activity and sleep quality among students with compromised physical health ought to be a cornerstone of mental health interventions, ensuring tailored support strategies for this susceptible group.

## Data Availability

The original contributions presented in the study are included in the article/supplementary material, further inquiries can be directed to the corresponding author.
